# The Effect of Different Nanomaterials Additions in Clay-Based Composites on Electromagnetic Transmission

**DOI:** 10.3390/ma15155115

**Published:** 2022-07-22

**Authors:** Ivan Vrdoljak, Jelena Brdarić, Slavko Rupčić, Berislav Marković, Ivana Miličević, Vanja Mandrić, Damir Varevac, Dalibor Tatar, Nikolina Filipović, Imre Szenti, Ákos Kukovecz

**Affiliations:** 1 Faculty of Civil Engineering and Architecture Osijek, Josip Juraj Strossmayer University of Osijek, Vladimira Preloga 3, HR-31000 Osijek, Croatia; ivana.milicevic@gfos.hr (I.M.); dvarevac@gfos.hr (D.V.); 2 Department of Chemistry, Josip Juraj Strossmayer University of Osijek, Cara Hadrijana 8/A, HR-31000 Osijek, Croatia; bmarkovi@kemija.unios.hr (B.M.); dtatar@kemija.unios.hr (D.T.); nfilipovic@kemija.unios.hr (N.F.); 3 Faculty of Electrical Engineering, Computing and Information Technologies Osijek, Josip Juraj Strossmayer University of Osijek, Kneza Trpimira 2B, HR-31000 Osijek, Croatia; slavko51062@gmail.com (S.R.); vmandric@gmail.com (V.M.); 4 Interdisciplinary Excellence Centre, Department of Applied and Environmental Chemistry, University of Szeged, Rerrich Béla tér 1, H-6720 Szeged, Hungary; szentiimre@gmail.com (I.S.); kakos@chem.u-szeged.hu (Á.K.)

**Keywords:** electromagnetic radiation shielding, clay composite, transmission, nanomaterials

## Abstract

In this study, clay composites were subjected to electromagnetic transmission testing at frequencies in the region of non-ionizing radiation. Specimens were made with partial substitution of clay with different admixtures by mass. Admixtures used were Fly Ash, four different particle sizes and phases of Titanium Dioxide (TiO_2_), Zinc Ferrite (ZnFe_2_O_4_), Maghemite (γ-Fe_2_O_3_) and Antimony Tin Oxide (ATO). The additives were thoroughly (chemically, structurally, morphologically) characterized. The replacement percentage was 5 wt.%. Electromagnetic transmission assessment included S_21_ transmission coefficient measurements for samples with different additives. The lowest transmission was reported for the clay specimens with ATO and Titanium Dioxide, especially at higher frequencies. A decrease in the transmission parameter with increasing specimen thickness was also confirmed.

## 1. Introduction

Electromagnetic (EM) radiation has been around much longer than humankind. The most common form of EM radiation for humankind is light. Nowadays, people are surrounded every day by a complex mix of electric and magnetic fields. Due to the rapid development of new technologies, year by year, new applications of EM fields are being adopted. It is almost impossible to imagine a year without a new application of EM fields for public use [1]. With regard to the increasing trend of human exposure to electromagnetic radiation, awareness of potential health problems also started to increase, especially from 1990 up to today [2]. In contrast to ionizing radiation, non-ionizing radiation, which this study will deal with, does not have enough energy to produce charged ions when passing through matter [3]. As a result, short-term exposure to low levels of non-ionizing radiation is not considered to be particularly harmful to people [4]. However, the effects of long-term exposure to non-ionizing radiation have not yet been completely explained. The scientific community is divided about the harmful effects of long-term exposure to this type of electromagnetic radiation on human health. According to the 2012 Bioinitiative report, between 2007 and 2012, there were approximately 1800 published studies that reported some effects due to human exposure to lower frequencies of non-ionizing EM radiation [5]. Due to the potential health risks, in 2011, the World Health Organization (WHO) classified radiofrequency (from 20 kHz to around 300 GHz) as possibly carcinogenic to humans. As the long-term effects have not yet been clearly determined, for precaution, there have been several methods devised to protect against non-ionizing EM radiation. In the past several years, the design of load-bearing structural elements (mostly concrete elements) with high shielding efficiency against EM radiation has been the subject of many investigations. A material acts as a shield against electromagnetic interference (EMI) when it restricts the penetration of electromagnetic fields into an inner space by reflecting or absorbing them with a barrier made of conductive material [6]. Because load-bearing elements, such as concrete or clay bricks have high electrical resistivity (concrete: 10^6^–10^9^ Ωm [7]; clay brick: around 10^6^ Ωm [8]), they cannot be used as EM shielding material. To reduce electrical resistivity, most often conductive fillers are added, such as carbon materials, steel fibers, steel slag, etc. [9,10,11]. In the available literature, no study was found that investigates clay bricks with improved electromagnetic shielding capabilities at non-ionizing frequencies. In two studies, the authors have investigated the electromagnetic shielding efficiency of clay bricks with the addition of Fly Ash, but their focus was on the γ-region (around 10^13^ Hz) [12,13] which belongs to ionizing radiation. In this study, the electromagnetic shielding efficiency of clay composites at a frequency range from 1.5 GHz to 6.0 GHz is investigated. At these frequencies, the electromagnetic radiation of mobile telephony systems can be found: LTE 1800 (1.80–1.88 GHz), LTE 2100 (2.11–2.17 GHz), LTE 2600 (2.62–2.69 GHz), and NR3500 (3.40–3.80 GHz). Various admixtures were used as clay fillers: Fly Ash, Zinc Ferrite (ZnFe_2_O_4_), four different types of Titanium Dioxide (TiO_2_), Maghemite (γ-Fe_2_O_3_), and Antimony Tin Oxide (ATO). All these admixtures were chosen because they have shown EM shielding capability when used in cementitious materials as a replacement for cement. Although Fly Ash is a waste material, its chemical composition contains Fe_2_O_3_ (usually up to 10 %), which can improve the shielding efficiency (SE) of the material due to its high electrical conductivity [14]. Zinc Ferrite belongs to spinel ferrites (CoFe_2_O_4_, MnFe_2_O_4_, ZnFe_2_O_4_, etc.). Due to their great electromagnetic properties and high permeability, the addition of Zinc Ferrite to the mortar resulted in higher reflectivity compared to the reference mortar [15]. Unlike ferrite materials, metal oxide materials, such as TiO_2_, although they are non-ferromagnetic, possess dielectric loss capacity. Therefore, they can also be considered as EM absorbents [16]. In the study by Li et al., 10 different types of TiO_2_ were used as a partial cement replacement in a cement composite. The results showed that all 10 types improved absorptivity with respect to the reference specimen [17]. Due to its good magnetic properties, the application of γ-Fe_2_O_3_ in materials and coatings has a positive effect on protection against electromagnetic radiation. This is confirmed by several studies that have reported an improvement in protection against EM radiation due to the addition of γ-Fe_2_O_3_ [18,19,20]. Although studies investigating ATO application in cementitious materials in the available literature have not been found, there have been reports of its good conductive properties that make it potentially suitable for shielding against electromagnetic radiation [21].

## 2. Materials and Methods

### 2.1. Materials

The commercially available nanomaterials used in this study were TiO_2_ Pretiox AV-01 FG obtained from Precheza (Přerov, Czech Republic), TiO_2_-A015, TiO_2_-A050, and TiO_2_-R050 from the division of MKNano-Canada. Fly Ash was procured from a supplier in Tuzla (Bosnia and Herzegovina). Antimony Tin Oxide Nanoparticles (ATO) were obtained from EPRUI (Shanghai, China), Maghemite (γ-Fe_2_O_3_) from IoLiTec (Heilbronn, Germany) and Zinc Iron Oxide (ZnFe_2_O_4_) from Nanografi (Ankara, Turkey). Clay was procured from the manufacturer Wienerberger (Đakovo, Croatia).

### 2.2. Methods

#### 2.2.1. Brunauer-Emmett-Teller Surface Area Measurements

The specific surface area of the materials was determined using B.E.T. Analysis on the Nova 4200e, Quantachrome© (Boynton Beach, FL, USA) instrument using the nitrogen ultra-high purity gas fill volume method. Firstly, the materials were degassed for 1 h at 350 °C and then analyzed at liquid nitrogen temperature.

#### 2.2.2. Powder X-ray Diffraction

Powder X-ray diffraction patterns were collected on the PANalytical Aeris Research Diffractometer (Malvern PANalytical, Malvern, UK) in θ-θ geometry, using monochromatized CuKα radiation (40 kV, 15 mA) at 295 K. Step size was 0.02, within a 2θ range from 20 to 100°.

#### 2.2.3. Measurements of Thermal Properties

The thermal properties of the materials were investigated using thermogravimetry (TGA/DSC) on Mettler Toledo System 1. The samples were heated in an oxygen atmosphere at a gas flow rate of 195 mL/min, heat rate of 10 °C/min in the temperature range of 50–1000 °C. The small amount of the materials (10–15 mg) was put in the open alumina (Al_2_O_3_) sample holders.

#### 2.2.4. Morphology Investigation

The surface structure and the composition of the samples was studied by the scanning electron microscopy (SEM) using the Thermo Fisher Scientific Apreo C instrument (Waltham, MA, USA) fitted with the Energy Dispersive X-Ray Analysis (EDX). The working distance was 10.3 mm, the applied voltage was 10 kV and 20 kV, and the spot size was 5 and 10 μm^2^.

#### 2.2.5. Mineral Composition and Heavy Metals Content

Determination of the oxide content was performed using an Energy Dispersive X-ray Fluorescence Spectrometer (ED-XRF). The manufacturer is Rigaku (Tokyo, Japan) instrument model NEX CG. The samples were previously sieved to a particle size of 50 µm and tableted on a manual hydraulic press Specac with a power of 15 tons for 30 s. The prepared tableted samples were then measured. Determination of annealing loss was performed with a Thermogravimetric Analyzer (TGA), manufacturer: LECO, model TGA 701. Heavy metal content in the ash was measured by Inductively Coupled Plasma Excitation Spectrometry (ICP-OES) on the Shimadzu (Kyoto, Japan) Model ICPE 9000. Samples were previously diluted by microwave digestion using the Anton Paar Multiwave PRO. Mercury content was determined using a mercury analyzer Perkin Elmer (Akron, OH, USA) model SMS 100.

### 2.3. Clay Test/Sample Preparation

**Figure 1:**
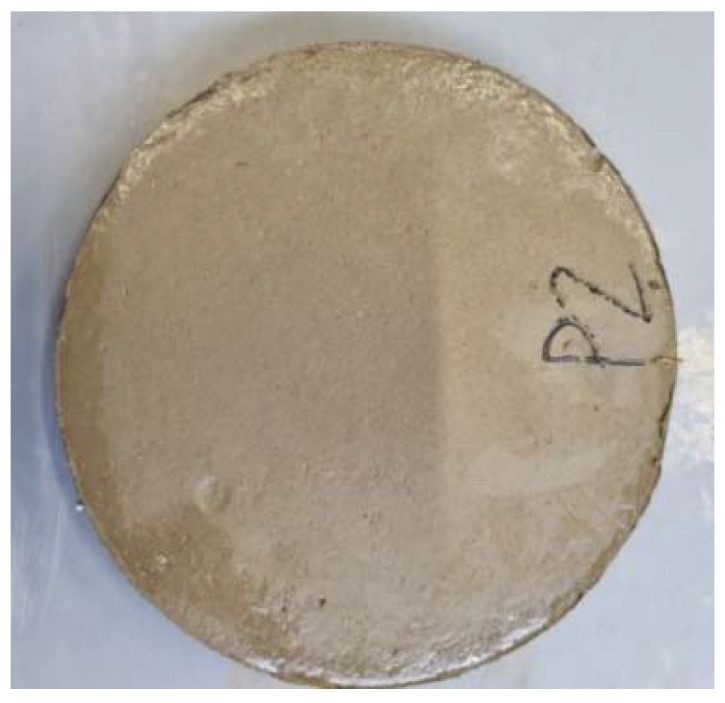
Fresh clay.

**Figure 2:**
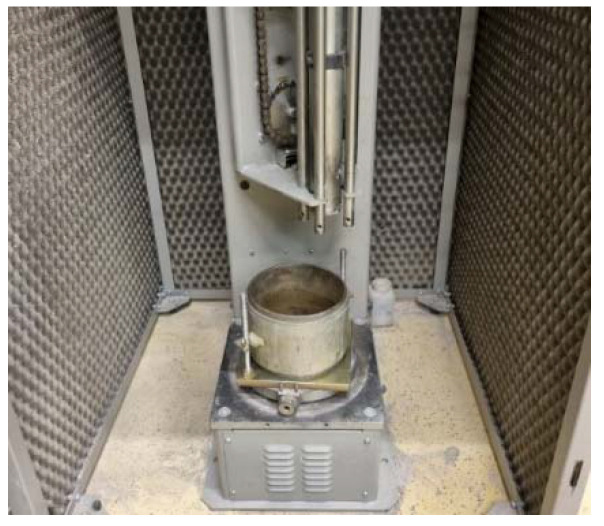
Sampling process.

**Figure 3:**
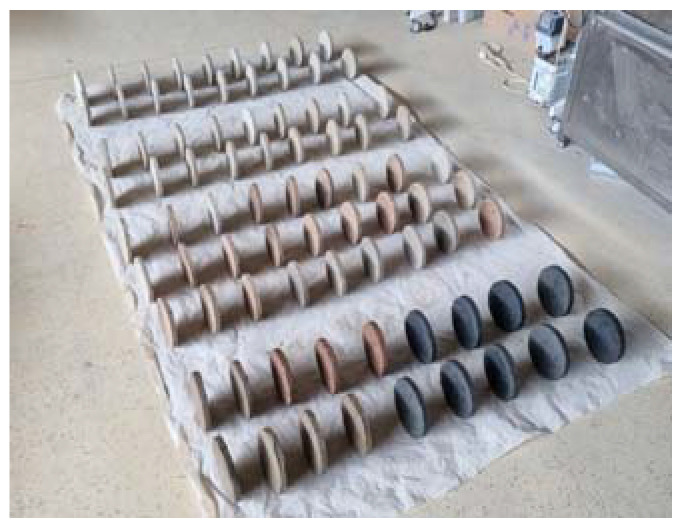
Air-drying of specimens.

**Figure 4:**
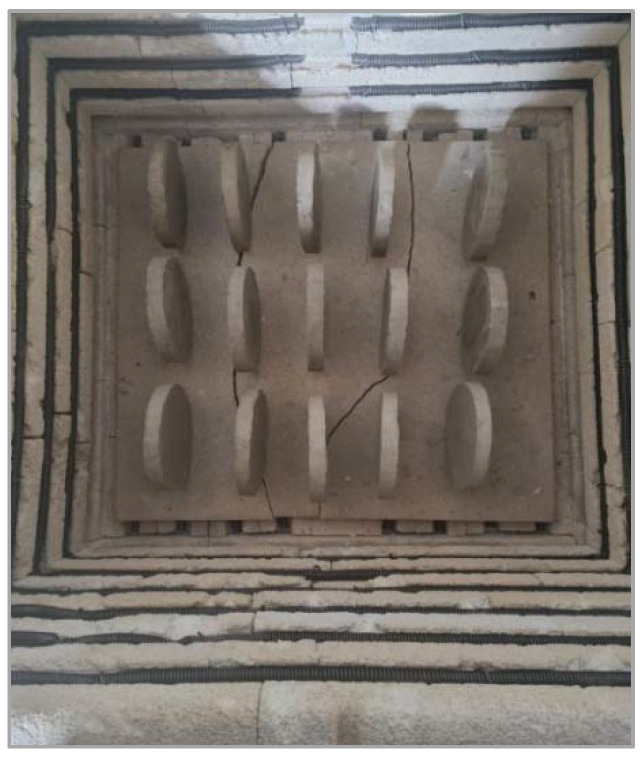
Firing process.

**Figure 5:**
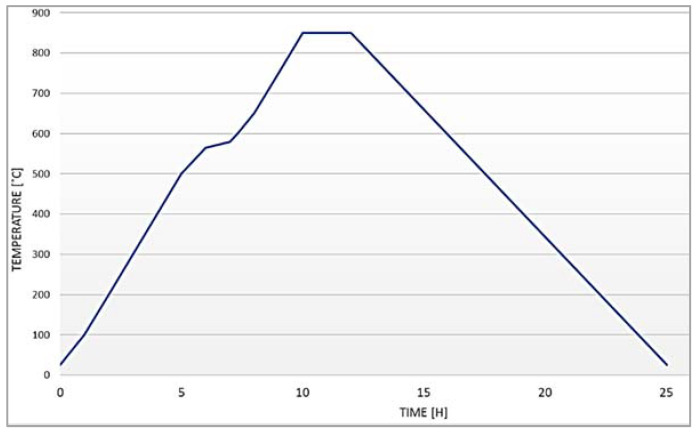
Firing program.

Raw clay was supplied from a local factory. For making clay composite specimens, clay was mixed and homogenized with admixtures in a dry state for 2 min. To achieve adequate workability, water was added to reach a moisture content of 20–22 wt.%. A total mixture of 650 g was prepared for each specimen. To ensure the validity of the results, 3 specimens were made for each mixture (Figure 1). The diameter of the clay cylinder specimens used for the measurement of EM shielding was 150 mm, while the thickness was 20 mm. To achieve equal mold pressure for every specimen, they were molded using a modified proctor. The mass of rammer was 4.5 kg and the height of fall was 457 mm. Every specimen was subjected to 30 rammer blows (Figure 2). The load was transferred equally to the sample using a 10 mm thick steel plate. The compaction energy was 1.71 MJ/m^3^. After molding, the specimens were dried. To avoid surface cracking, which can affect the results, the samples were dried in several steps: firstly, they were air-dried for two days at 70% relative humidity in a humidity-controlled room, after which they were air-dried for two more days at 30% relative humidity in the same space (Figure 3). The last step was drying in a laboratory oven at 105 °C for 24 h to completely remove water from the samples. Dried specimens were subjected to firing in an electric brick oven (Figure 4) after which specimens were ready for testing. The firing temperature used for all specimens was 850 °C. The samples were exposed to peak temperature for 3 h, while the overall firing process (including cooling) took 25 h. Figure 5 shows the heating profile. The firing program was supplied by the local brick factory, which is intended for firing load-bearing bricks.

## 3. EM Transmission Measurements

Electromagnetic radiation transmission was measured using the Anritsu ms2038c—Handheld Vector Network Analyzer and Spectrum Analyzer (Germany). Test frequency range was 1.5 GHz–6.0 GHz. Using Anritsu ms2038c S parameters, the shielding efficiency and the absorption-reflection coefficients of the samples with the admixtures were measured. A reference specimen (without any addition of admixture) was also measured. The measurement device contained two aluminum circular waveguides between which the measured specimen was placed in a steel plate.

Specimens were placed between waveguides using a steel plate, by which the specimen was fixed. To avoid any openings or slits between the specimen and the steel plate, aluminum foil was placed. All three elements (two waveguides and middle steel plate) were connected together using screws.

## 4. Results and Discussion

### 4.1. Properties of Nanomaterials

Before the preparation of samples and clay tests, commercially available chemicals were investigated by their qualitative and quantitative properties to confirm the credibility of nanomaterials. Powder X-ray diffraction (PXRD) was employed for confirmation of phase purity and specific surface area measurements were conducted to confirm the surface area and their comparison with apparent particle size. Morphology was investigated by SEM-EDX, while the qualitative composition was determined by X-ray fluorescence (XRF) and atomic spectroscopy (ICP-OES). Thermal properties of nanomaterials were investigated by thermogravimetry (TGA/DSC) to determine weight loss, phase changes, or the existence of surface moisture. Table 1 presents the results of the measured specific surface area of the materials using the nitrogen adsorption method according to B.E.T. and a comparison with particle size.

Thermograms (TGA) of various TiO_2_ samples measured in the temperature range from 25 to 1000 °C did not show any significant weight loss, Figure S1. The initial very small (0.6–2.1%) weight loss may be attributed to the presence of the moisture. The Clay sample shows a two-step weight loss at temperatures 50–160 °C and 350–650 °C, Figure S2 which would correspond to the loss of physically bound (step 1) and interlayer water from the clay structure (step 2). Additionally, as expected, samples of the Fly Ash, ATO, γ-Fe_2_O_3_ and ZnFe_2_O_4_ did not show substantial thermal loss in the specified temperature range (thermograms in Figure S3). The overall weight loss presented in Table 1 is mostly attributed to water, either on surface or in crystal structure.

Powder X-ray diffraction patterns of the TiO_2_ samples, both rutile and anatase, match the data from the Crystallographic Open Database (COD) for the same crystalline phases, except for the slight shifts in 2θ range, which is attributed to a reduction in particle size. Diffraction patterns of other materials (ATO, Fe_2_O_3_, and ZnFe_2_O_4_) also proved comparable to the data from the COD. Maxima corresponding to SiO_2_ were detected in the diffraction patterns of Fly Ash and Clay. All the diffraction patterns compared to the COD data are given in Figure S4.

**Figure 6:**
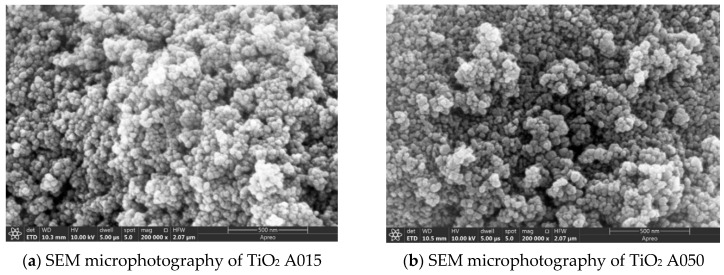

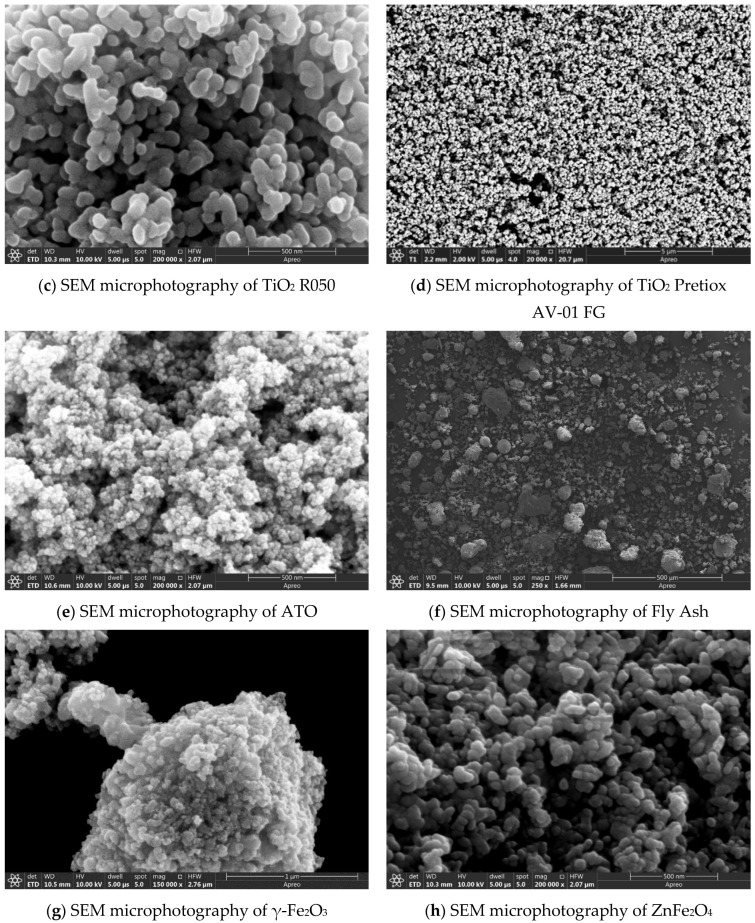
Scanning Electron Microscopy of clay additives (SEM).

Microphotographs of the materials are included in Figure 6 and are consistent with the data on particle sizes obtained from the manufacturer.

The elemental composition of the Fly Ash and the Clay samples is given in Table 2 (measured according to ISO/TS 16996: 2015). One can notice that the materials mostly have high Si content (52.41% and 71.52%), and Al (11.03% and 14.19%), respectively, which corresponds to their diffraction patterns. Table 3 shows the heavy metals content (in ppm) of the ash sample (according to the methods HRN EN ISO 16968.2015 and ASTM D6722-19 for Hg).

### 4.2. Measurements

Measurements of coupling parameters were performed on a series of brick samples with various additives that, according to the available literature, could have influenced the reduction in the EM wave transmission when passing through the sample. The following used additives are given in Table 4.

**Figure 7:**
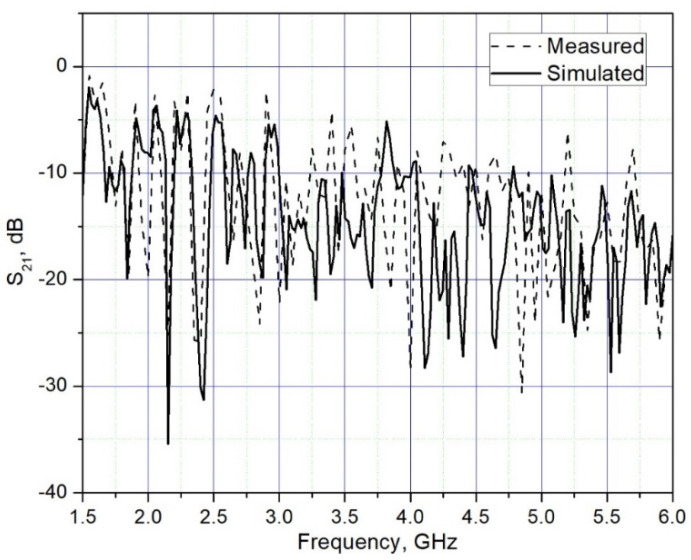
S_21_ measured and simulated results of brick sample in a frequency range 1.5 to 6 GHz, (d = 18 mm; 2r = 150 mm).

Beforehand, measurements of S parameters were performed for the reference sample (without additives) and these results were compared with the simulation results. The comparison shows a good match of the parameters, which indicates good agreement between the structural and EM parameters of the simulation model and the measured reference sample (Figure 7).

Measurement of the transmission parameter S_21_ was performed in the range from 1.5 to 6 GHz. Although the band frequencies below 1.5 GHz are also of interest, the current measurement system (based on waveguide EM wave transmission) does not allow the measurements at a lower frequency than reported. In the continuation of the research, measurements of S_21_ parameters will be performed also at the lower part of a spectrum.

**Figure 8:**
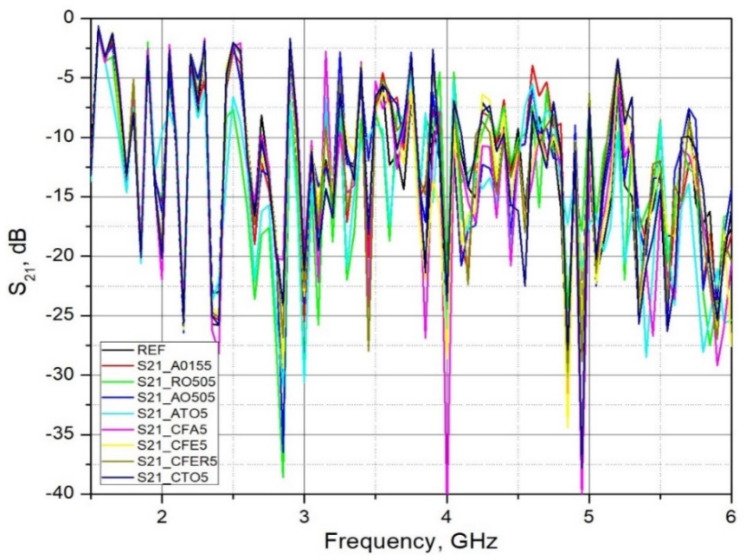
Measured S_21_ parameter of brick samples in a frequency range 1.5 to 6 GHz, (d = 18 mm; 2r = 150 mm).

**Figure 9:**
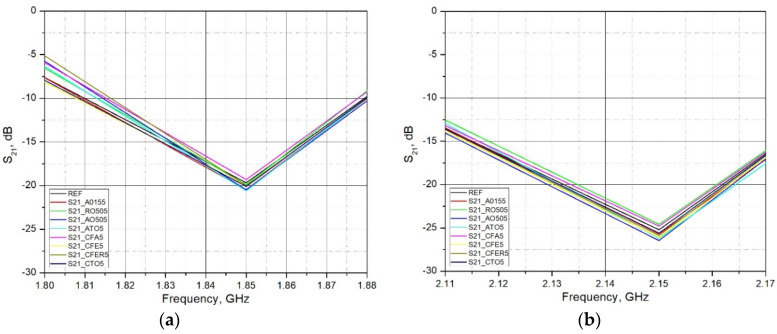
Measured S_21_ parameter of brick samples for (**a**) LTE1800 in a frequency range 1.8 to 1.88 GHz; and (**b**) LTE2100 in a frequency range 2.11 to 2.17 GHz, (d = 18 mm; 2r = 150 mm).

**Figure 10:**
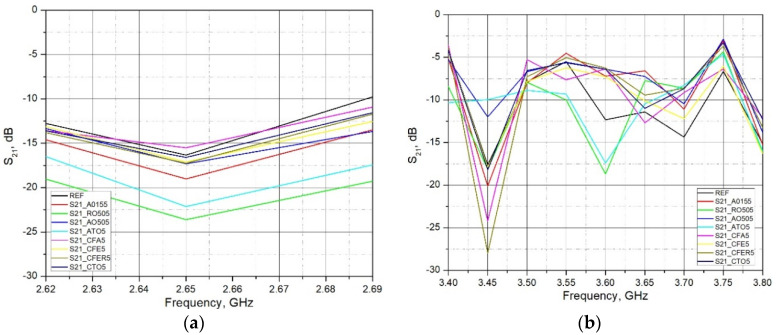
Measured S_21_ parameter of brick samples for (**a**) LTE2600 in a frequency range 2.62 to 2.69 GHz; and (**b**) NR3500 in a frequency range 3.40 to 3.80 GHz, (d = 18 mm; 2r = 150 mm).

Figure 8 shows the overall measured spectrum, while Figure 9 and Figure 10 show the parts of the spectrum that are of interest in this research, since these are the ranges of significant (in terms of radiation level) existing fixed radiation sources. These are the sources of the mobile telephony systems: LTE 1800, LTE 2100, LTE 2600 and NR3500 (5G system).

**Figure 11:**
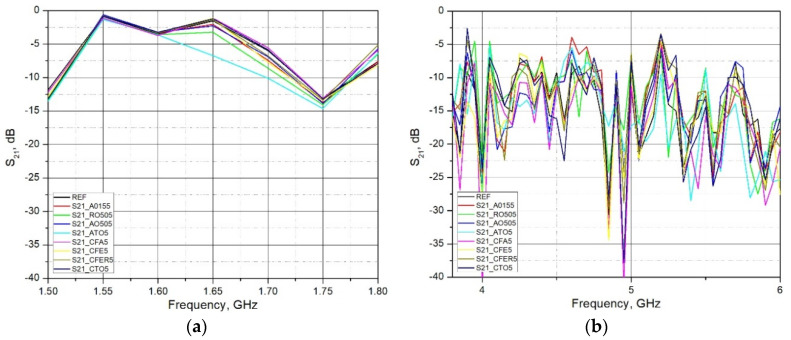
Measured S_21_ parameter of brick samples for (**a**) frequency range 1.50 to 1.80 GHz; and (**b**) frequency range 3.80 to 6.0 GHz, (d = 18 mm; 2r = 150 mm).

The ranges shown in Figure 11 are the remaining ranges covered by this measurement, so they need to be analyzed in addition to the previously selected ranges.

In the range from 3.7 to 5 GHz, there is a deviation of the measured and simulated values, and the reason is probably in the incomplete compliance of the parameters of the brick material used in the simulation and measurement.

### 4.3. Analysis of Test Results and Discussion

As expected, in most cases, all used admixtures showed the enhanced EM shielding behavior in comparison to the reference sample. As the frequency of EM wave transmission through the brick material increases, the S_21_ decreases (attenuation increases). The values of the S_21_ parameter at lower bands (LTE 1800 and LTE 2100) ranges from 2–5 dB, while at higher bands these differences are between 10–17 dB. Analyzing the frequency range 1.80 GHz–1.88 GHz (LTE1800) and 2.11 GHz–2.17 GHz (LTE 2100), it can be concluded that adding admixtures up to 5% by mass did not significantly lower transmission. The maximum increase was found to be around 5 dB. A more pronounced increase was reported in the frequency range 3.40 GHz–3.80 GHz (LTE 3500). A decrease in transmission was around 10–17 dB. Material with the addition of Titanium Dioxide (R0505P) and Antimony Tin Oxide (ATO5) in all measured ranges (except LTE2100) showed the lowest transmission (maximum attenuation) of the EM wave through the brick material (S_21_ lowest). The reason for the increase in the attenuation of admixtures may lie in the increased conductivity due to clay replacement with conductive materials. Although clay samples with the addition of Fe containing additives (ZnFe_2_O_4_, γ-Fe_2_O_3_, Fly Ash) in parts of the measured ranges show an increase in the attenuation of the EM wave, which indicates an increase in the electrical conductivity of the material, this increase cannot be considered as a significant one. The amount of these additives is probably too small to significantly increase the conductivity of the material and, thus, increase the attenuation of the EM wave.

## 5. Conclusions

This research analyzed the electromagnetic shielding capabilities of clay composites in the non-ionizing frequency range of 1.5 GHz–6 GHz. Admixtures that were used as partial clay replacement were: Fly Ash and four different types of TiO_2_, ZnFe_2_O_4_, γ-Fe_2_O_3_ and ATO. The special focus was on determining the transmission coefficient (S_21_). Experimental results of the reference sample showed little deviations in values in the range from 3.7 to 5.0 GHz. The reason for this deviation may lie in the probable incomplete compliance of the parameters of the brick material used in the simulation and measurement. Although a 1.5 GHz–6 GHz frequency span was analyzed, more focus has been placed on the specific areas, sources of mobile telephony systems: LTE 1800, LTE 2100, LTE 2600 and NR3500 (5G system). Analyzing the whole frequency span from 1.5 GHz to 6.0 GHz, not only frequencies that are used for mobile telephony systems, it can be concluded that the specimens with ATO (teal line) or TiO_2_ (green line) at most frequencies result in the lowest transmission.

The next step of the study will concern the analysis of the electromagnetic shielding efficiency of clay blocks with ATO and TiO_2_ as admixture, as well as increased values of relevant supplements. In addition, the research will expand to the spectrum below 1.5 GHz, which includes several other significant sources of electromagnetic radiation, such as mobile telephony base stations (2G, 3G, 5G at 700 MHz) and other fixed sources of electromagnetic radiation (DVBT2, FM, etc.). In the continuation of this study, it is necessary to include the reflection coefficients and thus complete the whole picture of EM wave propagation through different building materials.

## Figures and Tables

**Table 1:** Specific surface area (B.E.T.), particle size and thermal properties of the samples.

Nanomaterials	S_BET_/ m^2^g^−1^	Particle Size/ nm *	Weight Loss/ %
TiO_2_ Pretiox AV-01 FG	10.18	200	0.6–2.1
TiO_2_-A015	69.49	15	
TiO_2_-A050	64.39	50	
TiO_2_-R050	24.93	50	
Fly Ash	5.66	530 **	<6.0
ATO	59.63	50	
γ-Fe_2_O_3_	38.73	30	
ZnFe_2_O_4_	74.01	15	
Clay	39.42	***	^50−160°^ 4.4 ^350−650°^ 9.0

* Data provided by supplier, ** Calculated from the surface area, *** clay is delivered in blocks and crushed.

**Table 2:** Elemental composition of the fly ash and clay samples.

FLY ASH		CLAY	
Element	Value/Mass. %	Element	Value/Mass. %
P	<0.01	P	<0.01
Na	0.59	Na	0.89
K	1.38	K	2.12
Ca	20.44	Ca	4.05
Mg	3.76	Mg	1.98
Al	11.03	Al	14.19
Ti	0.67	Ti	1.36
Fe	6.83	Fe	3.79
Si	52.41	Si	71.52
Mn	0.22	Mn	0.02
S	2.66	S	0.06

**Table 3:** Heavy metal content of the fly ash sample.

Heavy Metal	Value/ppm
Pb	22.50
Cd	8.13
As	2.81
Ni	1145.0
Hg	0.071
Cr	620.50
Mn	1015.50
Co	33.80

**Table 4:** Used additives and label used for them further in text.

Additive	Label
Titanium dioxide	(A0505)
Titanium dioxide	(R0505)
Titanium dioxide	(A0155)
Titanium dioxide	(CTO5)
Antimony Tin Oxide	(ATO5)
Fly Ash	(CFA5)
γ-Iron (III) oxide	(CFE5)
Zinc Ferrite	(CFER5)

## Data Availability

Data is available from the corresponding authors on demand.

## Supplementary Materials

The following supporting information can be downloaded at: https://www.mdpi.com/article/10.3390/ma15155115/s1, Figures S1–S3, Thermogravimetric analysis of nanomaterials, Figure S4, Diffraction patterns of: (a) TiO2 A015, (b) TiO2 A050, (c) TiO2 R050, (d) TiO2 Pretiox AV-01 FG, (e) ATO, (f) Fly ash and clay, (g) γ-Fe2O3, (h) ZnFe2O4, compared to patterns from Crystallographic Open Database (COD) with their COD ID in legend.
