# Supplementary material for: The Effect of Different Nanomaterials Additions in Clay-Based Composites on Electromagnetic Transmission
Source: Materials (Basel). 2022 Jul 22;15(15):5115. doi: 10.3390/ma15155115 (PMC9330469; doi:10.3390/ma15155115)
Supplement: Supplementary file 1 [file materials-15-05115-s001.zip › materials-1799119-supplementary.pdf]

The Effect of Different Nanomaterials Additions in Clay-Based Composites on the Electromagnetic Transmission

Ivan Vrdoljak [1*], Jelena Brdarić [2*], Slavko Rupčić [3], Berislav Marković [2], Ivana Miličević [1], Vanja Mandrić [3], Damir Varevac [1], Dalibor Tatar [2], Nikolina Filipović [2], Imre Szenti [4], Ákos Kukovecz [4]

[1] Josip Juraj Strossmayer University of Osijek, Faculty of Civil Engineering and Architecture Osijek, Vladimira Preloga 3, HR-31000 Osijek, Croatia; ivrdoljak15@gfos.hr (I. Vrdoljak), ivana.milicevic@gfos.hr (I. Miličević), dvarevac@gfos.hr (D. Varevac)

[2] Josip Juraj Strossmayer University of Osijek, Department of Chemistry, Cara Hadrijana 8/A, HR-31000 Osijek; jbrdaric@kemija.unios.hr (J. Brdarić), bmarkovi@kemija.unios.hr (B. Marković), dtatar@kemija.unios.hr (D. Tatar), nfilipovic@kemija.unios.hr. (N. Filipović)

[3] Josip Juraj Strossmayer University of Osijek, Faculty of Electrical Engineering, Computing, and Information Technologies Osijek, Kneza Trpimira 2B, HR-31000 Osijek, Croatia; slavko51062@gmail.com (S. Rupčić), vmandric@gmail.com (V.Mandarić)

[4] Interdisciplinary Excellence Centre, Department of Applied and Environmental Chemistry, University of Szeged, Rerrich Béla tér 1, H-6720 Szeged, Hungary; szentiimre@gmail.com (I. Szenti), kakos@chem.u-szeged.hu (A. Kukovecz)

* Correspondence: ivrdoljak15@gfos.hr (I.Vrdoljak), jbrdaric@kemija@unios.hr (J.Brdarić)

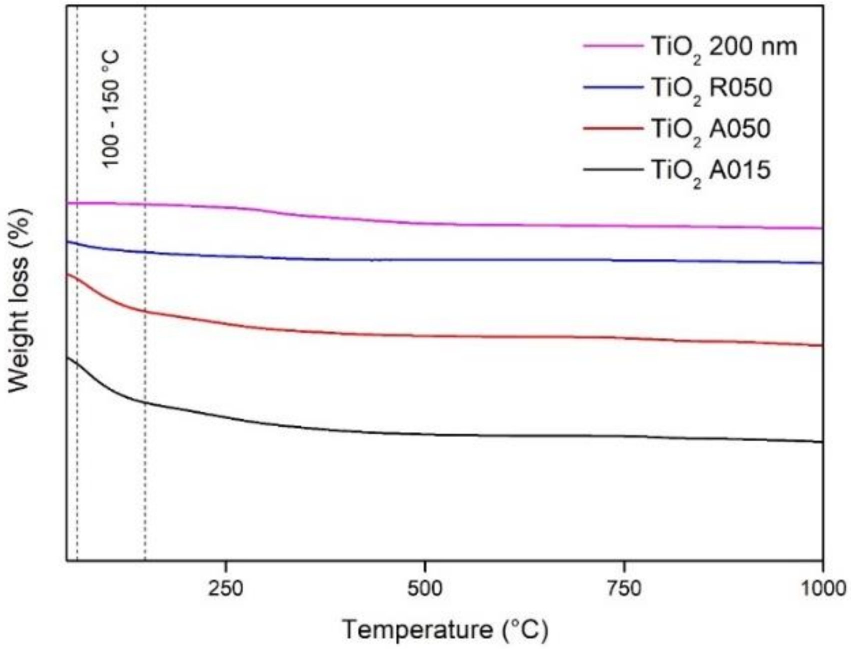

Figure S1. Thermograms of the four TiO_2 samples

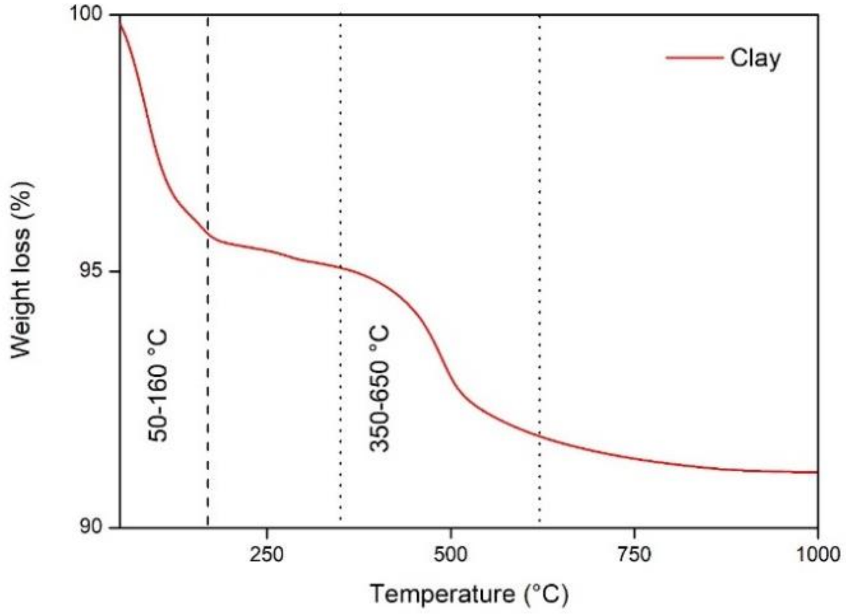

Figure S2. Thermogram of the clay sample

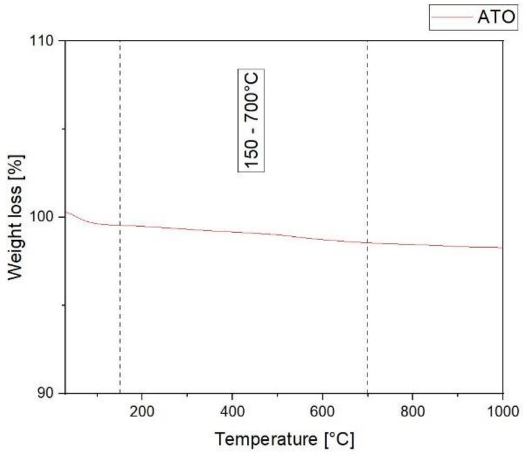

a) Thermogram of the Fly ash

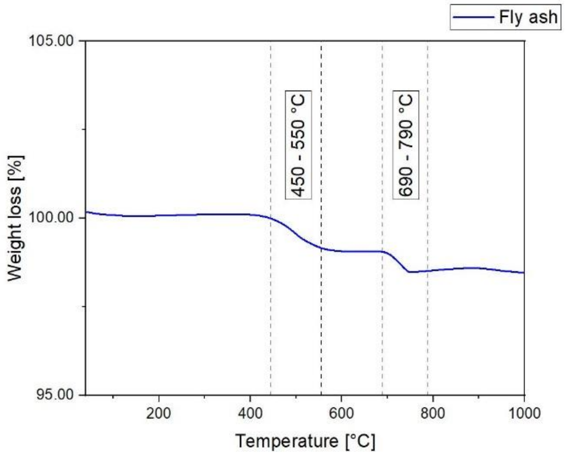

b) Thermogram of the ATO

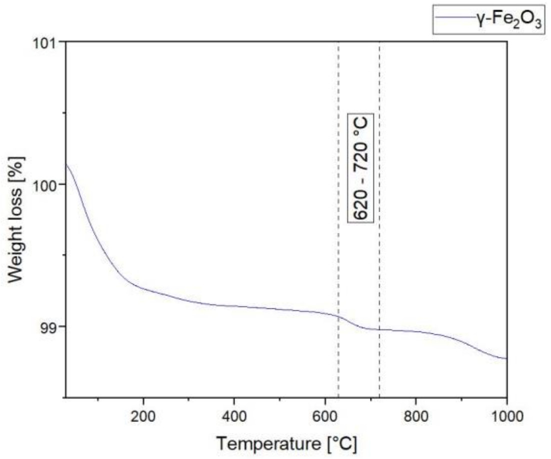

c) Thermogram of the γ-Fe_2O_3

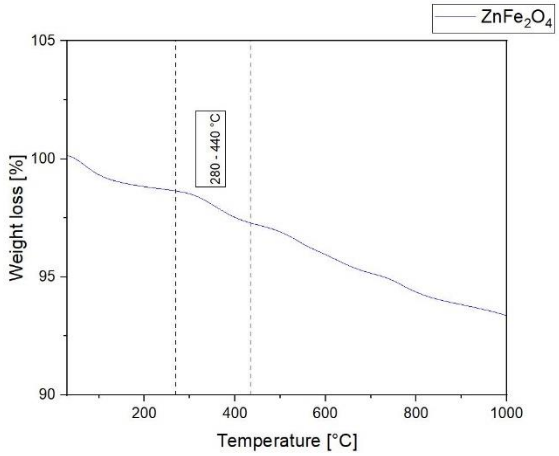

d) Thermogram of the $ZnFe_2O_4$

Figure S3: Thermogravimetric analysis of nanomaterials.

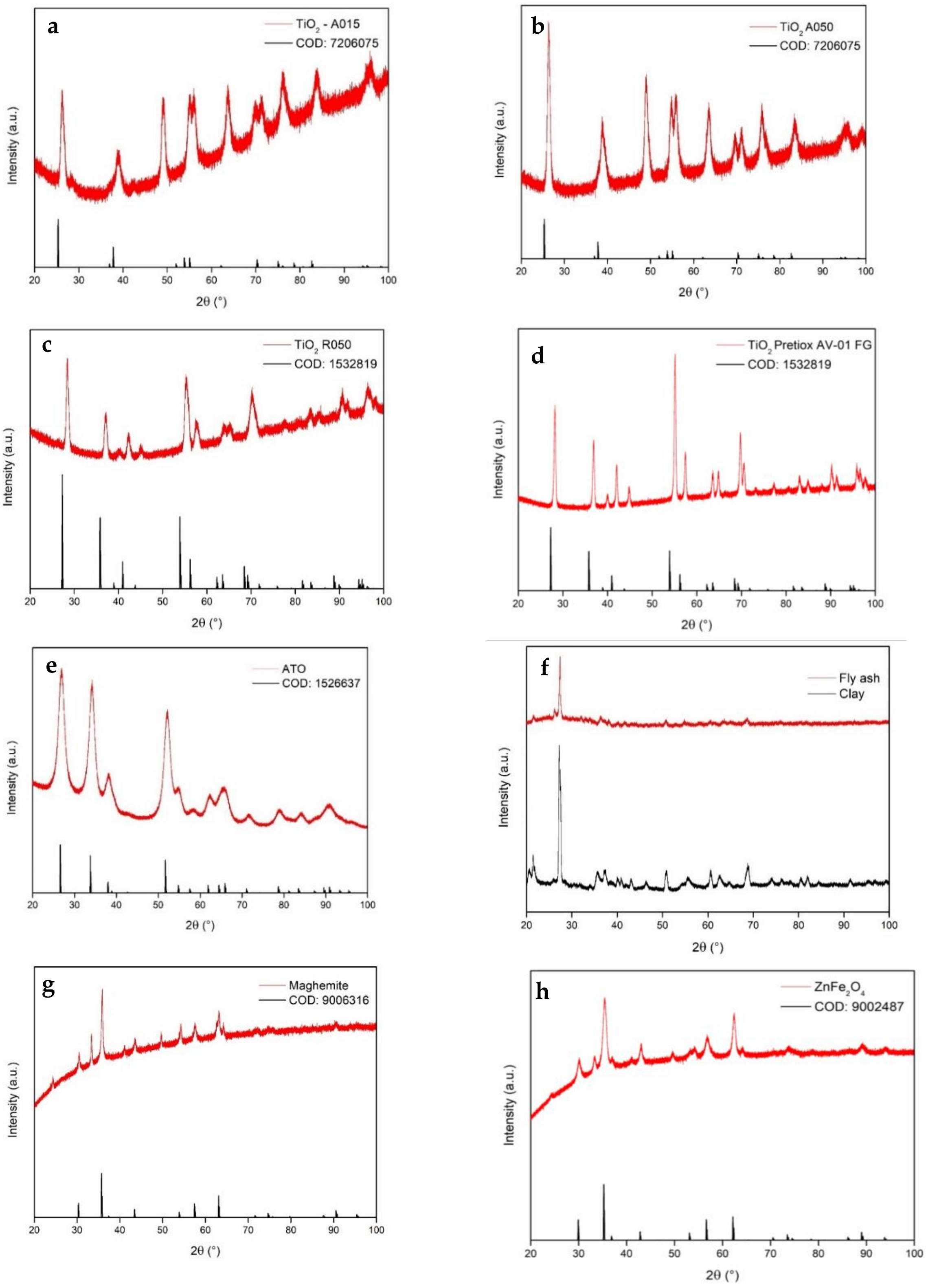

Figure S4: Diffraction patterns of: (a) TiO_2 A015, (b) TiO_2 A050, (c) TiO_2 R050, (d) TiO_2 Pretiox AV-01 FG, (e) ATO, (f) Fly ash and clay, (g) γ-Fe_2O_3, (h) $ZnFe_2O_4$, compared to patterns from Crystallographic Open Database (COD) with their COD ID in legend.
